# P270: Reduce maternal deaths related to the maternity hospital acquired infections: impossible pari for the Democratic Republic of the Congo?

**DOI:** 10.1186/2047-2994-2-S1-P270

**Published:** 2013-06-20

**Authors:** O Wembonyama, ML Tshilolo, C Tshintshiompo, WC Mpoy, KA Mutombo, MC Watu

**Affiliations:** 1 University of Lubumbashi, University Mbujimayi Muya, The Democratic Republic of the Congo; 2 CEFA, Munkole Hospital, Kinshasa, The Democratic Republic of the Congo; 3 University Institute of Congo, Lubumbashi, The Democratic Republic of the Congo

## Introduction

The safety of patients is not yet in the priorities of the health system in DRC despite the country is with Nigeria and Ethiopia, the head of the African countries that are at high risk (45, 410-2) of complications and maternal mortality against 1.34 10-4 in developed countries. Under these conditions, achieve the Millennium Development Goals to reduce by 2/3 of maternal mortality in 2015 is impossible, unless efforts are made in huge areas.

## Objectives

To assess the proportion of hospital infections in maternal morbidity and mortality observed in the maternity Lubumbashi.

## Methods

Descriptive cross-sectional study conducted in two maternity second (10 deliveries / day) and third levels (30 births / day) of the city of Lubumbashi. The relevant parameters are maternal mortality, hospital hygiene, quality of care, performance of biomedical laboratories, supplies of antibiotics.

## Results

It was noted:

In terms of hygiene: sanitary conditions are deplorable with no water points, disinfectants, antiseptics or gloves (unarmed deliveries). In terms of patient safety: No respects procedures during blood transfusions, infusions, injections, often performed cesarean section without asepsis emergency. In epidemiological terms: maternal mortality rate higher in the maternity tertiary level, especially related to the intra-hospital infections after cesarean section despite a higher skill level.

## Conclusion

Improving patient safety in maternity services in DR Congo through the implementation of emergency measures post-crisis to address issues related to the disruption of the health system, the morale and training of health personnel, and the paucity of hospitals.

## Competing interests

None declared

